# Vesico - Vaginal Fistula of Four Weeks’ Standing

**Published:** 1859-08

**Authors:** Ellery P. Smith

**Affiliations:** One of the Attending Surgeons of the Buffalo Hospital of the Sisters of Charity


ART. II.—*Vesico-Vaginal Fistula of Four Weeks' Standing. Operation and Cure.* By ELLERY P. SMITH, M. D., one of the Attending Surgeons of the Buffalo Hospital of the Sisters of Charity.

Mrs. C., æt. 40, twice married, and has had ten children. With her last, was in labor forty-eight hours, brow presentation; head arrested and impacted. Ineffectual means were resorted to, to change the position of the head; and perforation and extraction were had recourse to as a last resort. The delivery was effected March 12, 1859. Slight symptoms of hysteritis supervened, but yielded promptly to the usual remedies. Retention of urine existed for nearly a week, and the catheter was used daily. About ten days subsequent to the delivery of the fœtus, a detached slough of considerable size appeared, protruding through the vulva, and on being removed, was followed by a continuous flowing of urine, from the vagina. She entered the Buffalo Hospital of the Sisters of Charity, about the 1st of April, as a private patient. On making an examination, a vesico-vaginal fistula was at once detected, of about one inch in diameter, occupying the *bas fond* of the bladder, and situated about three and one half inches from the vulva. The patient's general health is tolerably good, although she suffers greatly from the excoriated condition of the thighs and nates; she has also a large external hemorrhoid. On April 21st, 1859, I performed the operation so ably described by Dr. Marion Sims, in the American Journal of Medical Sciences for the month of January, 1852. The operation was somewhat tedious, and some slight trouble was experienced from the twisting of the silver wire used with the clamps, owing to its not being properly annealed. Three sutures were used, and the lead clamps were nearly two inches in length. The hemorrhage was trifling, and the patient complained but little during the operation. The self-sustaining catheter of Dr. Sims was then introduced, and the patient placed in bed.

May 1st, I removed the clamps from the vagina, but continued the use of the catheter. During the ten days that have elapsed since the operation, she has been very comfortable, and, beyond a tympanitic condition of the bowels, which was relieved by injections, she has had no untoward symptoms whatever. About one week subsequent to the removal of the clamps, she left the hospital entirely well, being able to retain her urine as well as ever.

June 25th, 1859. She is now living with her husband, in the enjoyment of perfect health. In closing the report of this case, I cannot speak too highly of the instruments employed by Dr. Sims in this operation, than which nothing can be more simple and effectual; also of the position of the patient, and the use of the wide-guttered speculum, rendering an operation before so difficult of performance, one of the most easily executed in surgery.

